# Structural effects of whole body electromyostimulation on knee osteoarthritis: the EMSOAT Study

**DOI:** 10.1007/s00256-025-04984-5

**Published:** 2025-07-22

**Authors:** Frank W. Roemer, Stephanie Kast, Wolfgang Kemmler, Jamie E. Collins, Klaus Engelke, Ali Guermazi, Michael Uder, Simon von Stengel

**Affiliations:** 1 Department of Radiology, Universitätsklinikum Erlangen & Friedrich-Alexander-Universität Erlangen-Nürnberg (FAU), Maximiliansplatz 3, 91054 Erlangen, Germany; 2 Quantitative Imaging Center, Department of Radiology, Boston University School of Medicine, 820 Harrison Avenue, FGH Building, 4820 Harrison Avenue, FGH Building, 4th floor, Boston, MA 02118 USA; 3 Institute of Medial Physics, Friedrich-Alexander-Universität (FAU) Erlangen-Nürnberg, Henkestr. 91, 91052 Erlangen, Germany; 4 Orthopaedic and Arthritis Center for Outcomes Research (OrACORe) and Policy and Innovation EValuation in Orthopaedic Treatments (PIVOT) Center, Department of Orthopaedic Surgery, Brigham and Women’s Hospital, Harvard Medical School, 75 Francis Street, BTM Suite 5016, Boston, MA 02115 USA; 5 Department of Rheumatology & Immunology, Universitätsklinikum Erlangen & Friedrich-Alexander-Universität (FAU) Erlangen-Nürnberg, Ulmenweg 18, 91054 Erlangen, Germany; 6 Department of Radiology, VA Boston Healthcare System, 1400 VFW Parkway, Suite 1B105, West Roxbury, MA 02132 USA

**Keywords:** MRI, Knee osteoarthritis, Whole body electromyostimulation, MOAKS, Clinical trial

## Abstract

**Objective:**

Whole-body electromyostimulation (WB-EMS) might be an alternative option to conventional strength training for patients with knee osteoarthritis (OA). Our aim was to compare structural changes of knee OA between patients treated with WB-EMS and a control group (CG) treated with a standard of care approach.

**Material and methods:**

Seventy-two overweight participants with symptomatic knee OA were assigned to either WB-EMS or CG. MRIs were acquired at baseline and 7 months using a 3 T system. MRIs were read according to the MRI Osteoarthritis Knee Score (MOAKS) instrument. Between-group differences in regard to change in cartilage, bone marrow lesions (BMLs), osteophytes, meniscus damage and extrusion, and markers of inflammation were analyzed using Fisher’s Exact and Wilcoxon Rank Sum tests.

**Results:**

Fewer knees in the WB-EMS groups showed cartilage worsening compared to the CG (18% vs. 40%, *p* = 0.046). There were fewer knees in the WB-EMS group showing an increase in BML size score of ≥ 1 (30% vs 46%, *p* = 0.43). Regarding prevalent BMLs that showed improvement, no change, or worsening, no differences were seen (*p* = 0.56). Little osteophyte and meniscal changes were observed during the observational period. Differences regarding change in inflammatory markers were not significant.

**Conclusion:**

While there was a difference with fewer subregions showing worsening cartilage damage in the WB-EMS group, no significant differences were observed for change in BMLs, inflammation, osteophytes, and meniscal parameters. The observed improvement in clinical outcome parameters in favor of the WB-EMS group is likely due to other effects than improvement or less worsening of structural changes.

**Supplementary Information:**

The online version contains supplementary material available at 10.1007/s00256-025-04984-5.

## Introduction

Knee osteoarthritis (OA) is a major contributor to global disability [1]. The personal and socioeconomic impacts of knee OA are significant and are projected to rise in the coming decades, particularly due to the obesity epidemic [2]. Since there is currently no cure for OA, clinical guidelines focus on symptom management and functional improvement through interventions such as exercise, weight reduction and patient education [3, 4]. Various exercise regimens, including resistance and endurance training, have demonstrated positive effects on pain relief and functional improvement in individuals with knee OA [5]. A systematic review found that resistance training effectively reduced pain and/or improved daily functional abilities, with moderate to large effect sizes [6]. Overweight and obesity are key risk factors for the onset and progression of knee OA [7, 8], not only due to increased mechanical stress but also because visceral fat contributes to pro-inflammatory states that exacerbate OA [9].

Despite strong evidence supporting the benefits of physical activity and exercise for knee OA, most individuals with the condition do not meet recommended physical activity levels [10]. This may be due to a cycle of pain, avoidance of activity, decreased muscle strength, and further functional decline. Consequently, barriers to participating in resistance training to improve muscle strength are common. Whole-body Electromyostimulation (WB-EMS) offers an alternative to traditional resistance exercises. WB-EMS involves the simultaneous activation of multiple muscle groups in the trunk and limbs through electrical impulses delivered via specialized suits equipped with surface electrodes. Previous studies have shown WB-EMS to improve muscle strength, muscle morphology, and reduce fat mass in healthy individuals as well as those who are sarcopenic or functionally impaired [11–14]. The activation of large muscle groups through WB-EMS has also been linked to potential anti-inflammatory effects, contributing to its overall health benefits [15]. We could show previously in a randomized controlled trial focusing on knee OA that WB-EMS is effective in alleviating pain, objective lower-limb function and maximum strength of hip-/leg extensors compared to a usual care approach [16]. Whether WB-EMS also has concurrent positive effects on joint structure and whether these observed clinical improvements are associated with concomitant improvement in structural changes is not known.

Thus, the aim of our study was to compare structural changes of knee OA as assessed by semi-quantitative magnetic resonance imaging (MRI) assessment between patients with knee OA treated with WB-EMS and those treated with an OA-specific standard of care approach over a period of 7 months. A secondary aim of this analysis was the evaluation of changes in structural parameters following the interventional period from month 7 to month 12.

## Methods

### Study design

The EMSOAT trial (Whole-Body Electromyostimulation for the Treatment of Knee Osteoarthritis) is a multicenter, parallel-group, randomized controlled trial (RCT) with a 1:1 allocation ratio, designed to assess superiority. The study is being conducted at the Institute of Medical Physics, Friedrich-Alexander University Erlangen-Nürnberg (FAU), and the Department of Radiology at University Hospital Erlangen, Germany. Ethical approval was granted by the FAU Ethics Committee (reference number 352_20 B), and all participants provided written informed consent prior to enrollment. The study adheres to the principles outlined in the Declaration of Helsinki and was prospectively registered on ClinicalTrials.gov (NCT05672264) on January 5, 2023.

### Participants

Participants were recruited between March and June 2022 from the metropolitan region of Erlangen-Nürnberg, Germany. Eligibility criteria included (1) male or female individuals aged 40 to 70 years; (2) overweight status, defined as a body mass index (BMI) > 25 kg/m^2^; (3) radiographically confirmed femorotibial osteoarthritis corresponding to Kellgren-Lawrence (KL) grades 2 or 3 (see detailed description below); (4) a history of knee pain persisting for at least 3 months; (5) knee pain present on at least 50% of the days within the past 30 days; and (6) an average pain intensity score > 2.5 on a 0–10 numeric rating scale (NRS). Exclusion criteria, previously published in detail, included contraindications to whole-body electromyostimulation (WB-EMS), recent trauma to the affected knee joint, or intra-articular injection within the preceding 3 months.

**Fig. 1:**
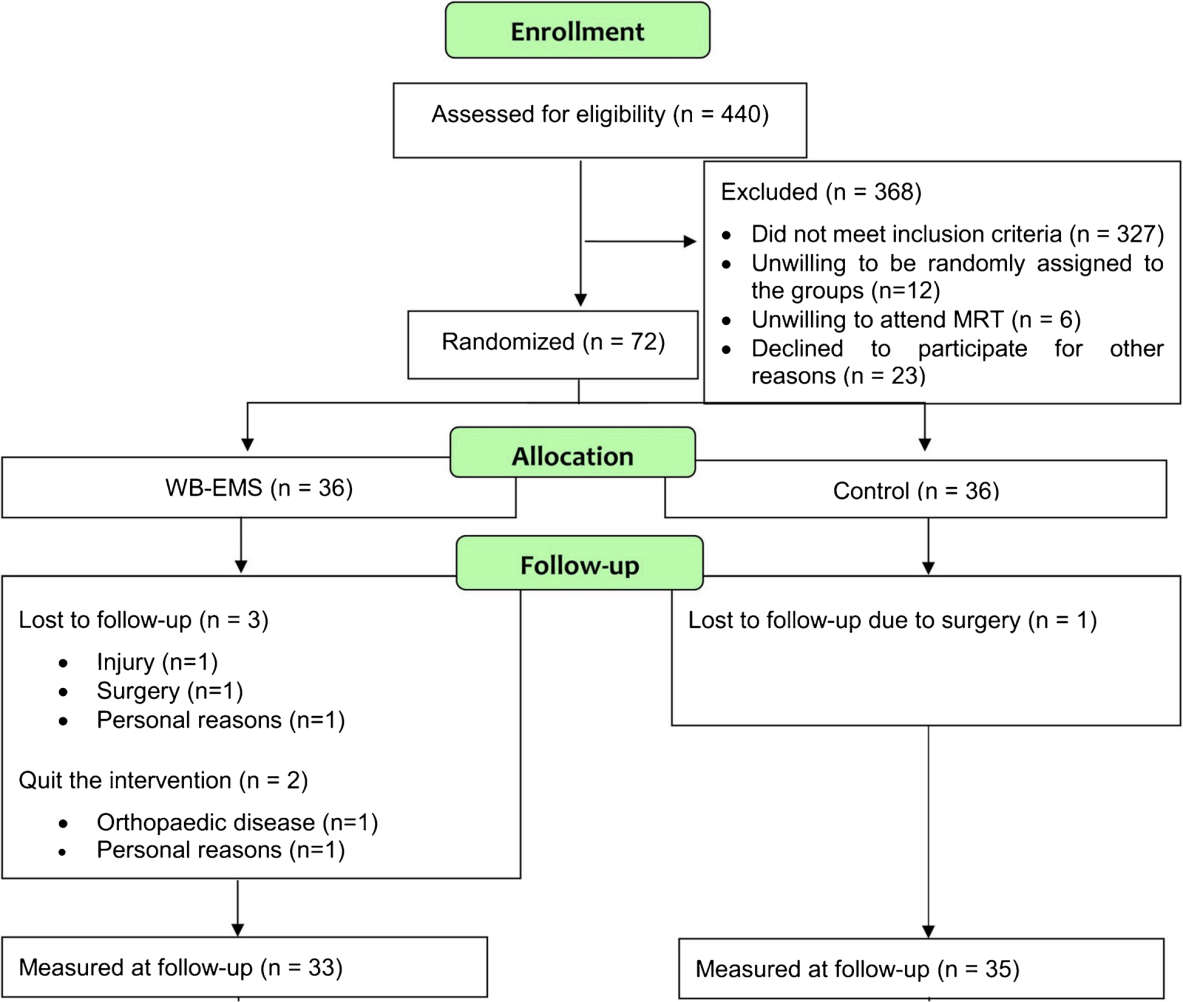
Flowchart of study inclusion

A flow chart of study inclusion is presented in Fig. 1. As radiographs could not be obtained for study purposes only due to legal constraints, potential participants were asked to provide externally acquired anterior–posterior radiographs of their index (more painful) knee when available. These were assessed by an experienced musculoskeletal radiologist (FWR) and those with KL 2 or KL 3 were included. Participants without externally acquired radiographs or radiographs older than 2 years were screened by MRI and those with full-thickness cartilage damage at both the femur and tibia in at least one compartment (grades 3.2 or 3.3 in at least one central femoral and one subregion of the anterior, central and posterior tibial subregions on the MRI Osteoarthritis Knee Score (MOAKS) scale were excluded [17]. Also, those with no or only focal cartilage damage (maximum of 1.0 or 1.1. in the 10 femoro-tibial subregions of the MOAKS instrument) were excluded. Using these MRI definitions, the likelihood of including KL 0 and 1 knees or knees with end stage structural OA (KL 4) was minimized. If both knees of a single participant were eligible, we defined the side that caused more pain as the “index limb.”

### Randomization and blinding procedures

Briefly, participants were randomly assigned to the WB-EMS and control group using the envelope method. In detail, each of the 72 eligible participants drew a small opaque capsule from 72 capsules that had been placed in a bowl by a researcher not involved in the trial. Thus, neither study participants nor researchers involved in the trial were aware of the allocation beforehand, and thus, respecting allocation concealment. Following the randomization procedure, participants were registered and instructed about the details of the study methodology. Blinding refers to researchers and research assistants that were unaware of the participants’ group status (WB-EMS or CG).

### Intervention

The WB-EMS group performed 1.5 times per week (once in one week, twice in the subsequent week) a WB-EMS training session of 20 min each for 7 months (including 1 month of conditioning), while the CG was provided with 6 × 20 min standard physiotherapeutic treatments including hands-on techniques and exercises. The WB-EMS group conducted a bipolar stimulation program with an 85 Hz (impulse frequency), 350 µs (impulse width), immediate impulse raise, applying a duty cycle of 6 s of impulse intermitted by 4 s of rest. During the impulse phase, low amplitude movements with minor voluntary effort were conducted. After 4 weeks of familiarization, participants were asked to exercise at a rate of perceived exertion of “6–7” (i.e. “hard + to very hard”) on the Borg CR10 scale [18]. This procedure can be considered as the standard WB-EMS protocol in research but also in commercial WB-EMS facilities [19]. Both groups completed a self-management program for OA of 6 sessions over 12 weeks including lectures in order to promote personal responsibility and develop coping strategies for dealing with pain and the disease knee OA in general. Details regarding the intervention in both groups have been published previously [16].

### MRI acquisition and assessment

MRIs were acquired at baseline and 7 months follow-up using a 3 T system (Siemens Magnetom Prisma, Erlangen, Germany). The protocol consisted of triplanar intermediate-weighted fat-suppressed and a coronal T1-weighted sequence without fat suppression. One musculoskeletal radiologist with 19 (FWR) years’ experience in SQ assessment of knee OA at the time of reading, blinded to all clinical data, performed the MRI assessment according to the MOAKS instrument with knowledge of the chronological order of the scans. The following joint structures were assessed: cartilage morphology, subchondral bone marrow lesions (BMLs), osteopyhtes, meniscal structural damage and meniscal extrusion, Hoffa-synovitis and effusion-synovitis.

In addition, within-grade changes were considered that fulfill the definition of a definite visual change but do not fulfill the definition of a full-grade change on the ordinal scales applied. Evaluation of within-grade changes increases sensitivity to change, has been shown to be clinically valid and is associated with concurrent quantitatively assessed cartilage loss [20, 21]. Within-grade changes were applied for cartilage and BML assessment. For cartilage, within-grade changes were coded for the area-extent dimension and the full-thickness dimension of the MOAKS scale, separately.

### Features assessed and change over time

#### Cartilage

MOAKS uses a two-digit score for cartilage assessment (each 0–3) that incorporates both area size per subregion (i.e., in the following referred to as “area extent”-dimension) and percentage of subregion that is affected by full-thickness cartilage loss (i.e., in this analysis referred to as “full-thickness”-dimension). Baseline frequencies for both cartilage dimensions are presented for maximum MOAKS score and number of subregions with any cartilage damage on a knee and on a compartmental level. The number of subregions showing any worsening (i.e., a higher score at month 7 compared to baseline) was defined for any change in MOAKS score, i.e. area extent and/or full-thickness or change in both. Change over time on a knee and compartmental level was defined as increase in number of subregions showing any MOAKS cartilage worsening including within-grade changes. Within-grade scoring for cartilage refers to any within-grade change in area or thickness for the total MOAKS score evaluations. In addition, change was categorized into none vs. any change.

#### BMLs

MOAKS assesses BMLs in three dimensions: % of subregion affected by any BML (0–3), % of subregion that is cystic vs. ill-defined BML (0–3) and number of BMLs per subregion. In this study, only the size component was considered. Change (including full grade and within-grade changes) in overall number of subregions affected by any BML was defined as the difference between the number of subregions affected by any BML at month 7 (size > 0) and the number of subregions affected by any BML at baseline. This was further categorized into overall improvement, no change, and worsening in one or more subregions. Further, the maximum increase in BML score from baseline to 7 months was determined on a knee and compartmental level.

#### Osteophytes

MOAKS scoring considers osteophytes at 12 possible marginal locations of the joint from 0 to 3. Change in maximum osteophyte score from baseline to month 7 was defined as the greatest amount of worsening of all affected locations per knee or compartment.

#### Meniscus

MOAKS evaluates meniscus damage from 0 to 8 with grade 1 representing intrameniscal signal but no tear or maceration. Grades 2–5 represent different tear types and grades 6–8 reflect maceration, i.e., meniscal substance loss. Furthermore, meniscal extrusion was scored in the anterior and mid-joint locations from 0 to 3. We assessed whether there was worsening in meniscal morphology from baseline to month 7 in any of the three medial or lateral meniscal subregions. These were assessed separately. We defined worsening as an increase in grade in at least one subregion. We assessed changes in meniscal extrusion separately in the medial and lateral compartments as any change vs. no change.

#### Hoffa-Synovitis and Effusion-synovitis

As MRI markers of inflammation, so-called effusion- and Hoffa-synovitis are evaluated in MOAKS. Hoffa-synovitis is a term used for signal changes in Hoffa’s fat pad that are commonly used as a surrogate for synovitis on non-contrast enhanced MRI. Effusion-synovitis is scored from 0 to 3 according to the distention of the joint capsule as 1 = small, 2 = moderate, and 3 = large. Hoffa-synovitis is scored based on the amount of hyperintensity signal in Hoffa’s fat pad on sagittal fat suppressed intermediate-weighted sequences as 1 = mild, 2 = moderate, and 3 = severe. Frequencies of baseline Hoffa- and effusion synovitis are presented. 7-month changes in both, Hoffa-synovitis and effusion-synovitis are assessed separately and categorized as improvement, no change, or worsening.

Identical definitions for change were applied for all features for changes from month 7 to month 12.

### Sample size and analytic approach

The sample size analysis was based on the primary clinical study endpoint of the EMSOAT study (KOOS-Pain [22]). Due to the lack of data on the effect of WB-EMS in OA, the power analysis was based on the effects of conventional strength training on pain in knee OA based on the meta-analysis by Goh et al. [23]. It is important to note that the power calculation did not address any of the outcomes used in the current imaging study but focused on the primary study endpoint of the EMSOAT project, i.e., the KOOS-Pain score. Applying a two-sided student t-test, the sample size of 36 subjects per group (WB-EMS: *n* = 36, UCG: *n* = 36) was selected to provide > 80% power (*α* = 5%) to detect a standardized mean difference between the WB-EMS and CG groups of 0.67. Two sided tests were applied, a *p*-value < 0.05 was considered statistically significant. Power calculation was performed using the statistical software R [24].

Between-group differences in regard to change from baseline to month 7 (= V1 to V2) and from month 7 to month 12 (= V2 to V3) for cartilage, bone marrow lesions (BMLs) (including within-grade changes for cartilage and BMLs), osteophytes, meniscus damage and extrusion, and markers of inflammation (Hoffa- and effusion synovitis) were analyzed using Fisher’s Exact and Wilcoxon Rank Sum tests. No multiplicity adjustments were applied to statistical testing, as these analyses are exploratory in nature and intended to be hypothesis generating [25, 26].

All analyses regarding the MRI outcomes were conducted using SAS 9.4 (SAS Institute, Cary NC).

## Results

Altogether 72 participants were included. Mean age of the participants in the WB-EMS group was 58.3 ± 7.2 years, mean BMI was 31.1 ± 4.6 kg/m^2^ and mean knee pain was 4.05 ± 1.45 (NRS scale 0–10 averaged over 7 days). No significant differences between the groups were observed for any of the baseline demographic values. In parallel, no baseline differences between the groups were observed for potentially confounding factors (e.g., physical activity, exercise volume, leg extension strength, knee pain and pain medication). However, considering a absolute standardized mean difference (SMD) higher than 0.2 as at least partly imbalanced, BMI (SMD = 0.43), body mass (SMD = 0.25) and isokinetic hip/leg extension strength (SMD: 0.21) differed between the groups.

Of the 72 subjects randomized, 4 subjects were lost to follow-up for reasons unrelated to the study (WB-EMS: *n* = 3; CG: *n* = 1) leaving 68 patients available for the MRI analyses (*n* = 32 for WB-EMS and *n* = 35 for the CG).

The groups were well balanced on baseline cartilage damage scores. Regarding baseline cartilage damage (area extent dimension) and on a knee level, mean number of subregions affected by any cartilage damage was 7.6 (SD ± 2.0) without differences between groups (7.7 CG vs. 7.5 WB-EMS; *p* = 0.62). Regarding area extent on a compartmental level, 63% of participants had a maximum score of 2 and 29% of 3 in the MFTJ, 41% of grade 2 and 12% of grade 3 in the LFTJ and 60% and 35% in the PFJ. Concerning the cartilage full-thickness dimension on a knee level the mean number of subregions affected at baseline was 3.5 (SD ± 1.7) without differences between groups (3.6 CG vs. 3.4 WB-EMS, *p* = 0.62). On a compartmental level 46% showed a maximum grade of 2, and 3% a maximum grade of 3 in the MFTJ while these numbers were 12% and 6% for the LFTJ and 37% and 9% for the PFJ, characterizing this cohort as mainly medial OA with or without PFJ involvement. An overview of baseline cartilage damage regarding area extent and full thickness component is shown in Appendix 1.

**Fig. 2:**
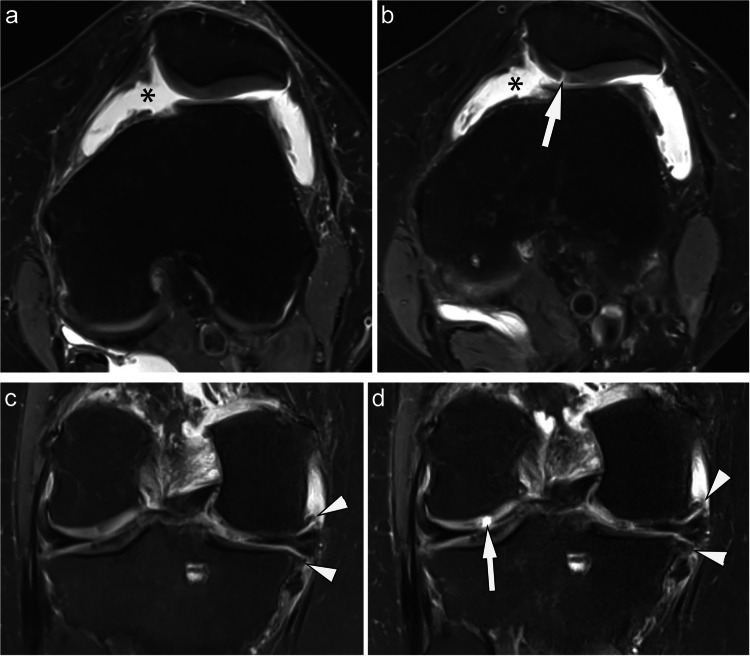
While overall little change was seen for cartilage, incident focal defects were seen in both treatment groups in some patients. **A**. Axial intermediate-weighted fat suppressed image shows an intact retropatellar cartilage surface without cartilage damage. There is marked joint effusion at the baseline visit (asterisk). **B**. At the 7-months follow-up visit, there is an incident focal full-thickness lesion (MOAKS grade 1.1) at the medial patella (arrow). There is persistent joint effusion (asterisk). **C**. another patient shows diffuse superficial medial cartilage damage and definite marginal osteophytes at the femur and tibia (arrowheads). **D**. At the 7 months follow-up visit, there is incident focal full thickness cartilage lesion at the central lateral femur (arrow). In addition, there is minor but definite increase in osteophyte size (arrowheads)

Concerning subregional change on a knee level from baseline to 7 months, fewer knees in the WB-EMS groups showed cartilage worsening (any subregions with worsening; area extent and/or full thickness dimensions including within-grade changes) compared to the CG (18% vs. 40%, *p* = 0.046). On a compartmental level, slightly more knees CG showed an increase in number of subregions showing worsening in the MFTJ compared to WB-EMS, with (14% vs. 6%), albeit not statistically significant (*p* = 0.67), and also in the LFTJ (11% vs. 3%, *p* = 0.35), but not in the PFJ (17% vs. 15%, *p* = 0.74). Details of these results are shown in Table 1. Illustrative examples of cartilage changes in the cohort are depicted in Fig. 2.

**Table 1:** Cartilage subregion worsening from V1 to V2 (change in area extent and/or full thickness dimensions including within-grade changes)

	**Overall** (*n* = 68)	**Control** (*n* = 35)	**WB-EMS** (*n* = 33)	***p*-value**
Number of subregions with worsening (including within-grade changes) V1 to V2 – Knee				
0	48 (71%)	21 (60%)	27 (82%)	0.05
1	17 (25%)	12 (34%)	5 (15%)	
2	2 (3%)	2 (6%)	0 (0%)	
4 +	1 (1%)	0 (0%)	1 (3%)	
Any subregions with worsening (including within-grade changes) V1 to V2 – Knee				
No	48 (71%)	21 (60%)	27 (82%)	0.06
Yes	20 (29%)	14 (40%)	6 (18%)	
Number of subregions with worsening (including within-grade changes) V1 to V2 – MFTJ				
0	61 (90%)	30 (86%)	31 (94%)	0.67
1	6 (9%)	4 (11%)	2 (6%)	
2 +	1 (1%)	1 (3%)	0 (0%)	
Any subregions with worsening (including within-grade changes) V1 to V2 – MFTJ				
No	61 (90%)	30 (86%)	31 (94%)	0.43
Yes	7 (10%)	5 (14%)	2 (6%)	
Number of subregions with worsening (including within-grade changes) V1 to V2 – LFTJ				
0	63 (93%)	31 (89%)	32 (97%)	0.36
1 +	5 (7%)	4 (11%)	1 (3%)	
Number of subregions with worsening (including within-grade changes) V1 to V2 – PFJ				
0	57 (84%)	29 (83%)	28 (85%)	0.74
1	10 (15%)	6 (17%)	4 (12%)	
2 +	1 (1%)	0 (0%)	1 (3%)	
Any subregions with worsening (including within-grade changes) V1 to V2 – PFJ				
No	57 (84%)	29 (83%)	28 (85%)	1.00
Yes	11 (16%)	6 (17%)	5 (15%)	

V1: baseline visit; V2: 7 month visit; MFTJ -medial femoro-tibial joint; LFTJ: lateral femoro-tibial joint; PFJ: patello-femoral joint; WB-EMS: whole body electromyostimulation

Fisher’s Exact test or Wilcoxon rank sum test was applied to calculate *p*-values

**Fig. 3:**
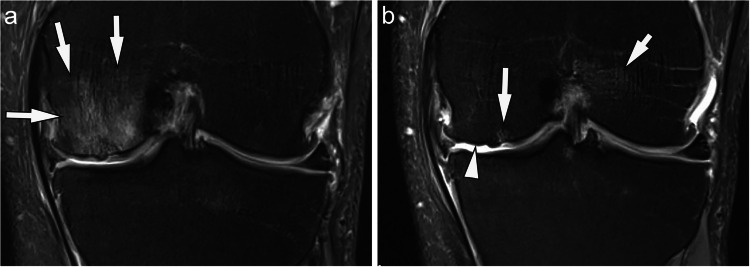
Change in bone marrow lesions (BMLs) from baseline to month 7. **A**. Baseline coronal intermediate-weighted fat suppressed image shows a large MOAKS grade 3 BML in the central medial femur subregion (arrows). In addition, there is superficial medial femoral and tibial cartilage damage. **B**. At 7-months follow-up, there is marked decrease in BML to now a small residual grade 1 lesion at the medial femur (long arrow). In addition, there is worsening cartilage damage at the femur with now diffuse full-thickness damage visible (arrowhead). A new, not cartilage–related bone marrow edema is seen at the femoral origin of the anterior cruciate ligament due to biomechanical traction phenomena (short arrow)

For change in BMLs no differences were seen between the EMS and CG for number of subregions showing worsening or improvement, which was also the case for the compartmental analyses. Twenty-nine percent of knees had fewer subregions with any BML from baseline to 7 months, 60% had the same number of subregions with BMLs, and 10% had more subregions with BMLs. This was similar between groups (*p* = 0.67). An example of BML improvement in a patient of the WB-EMS group is shown in Fig. 3. There were fewer knees in the WB-EMS group showing an increase in a BML size score in any subregion: 46% of participants in the CG had at least one subregion with worsening BML size score compared to 30% of WB-EMS participants, however, these findings were not statistically significant (*p* = 0.43). An overview of the BML results are shown in Table 2 and Appendix 2.

**Table 2:** Change in bone marrow lesions (BMLs) from V1 to V2—subregion approach

	**Overall** (*n* = 68)	**Control** (*n* = 35)	**WB-EMS** (*n* = 33)	***p*-value**
Change in subregions with BML V1 to V2—Knee				
Improvement	20 (29%)	12 (34%)	8 (24%)	0.67
Stable	41 (60%)	20 (57%)	21 (64%)	
Worsening	7 (10%)	3 (9%)	4 (12%)	
Change in subregions with BML V1 to V2—MFTJ (Category)				
Improvement	5 (7%)	3 (9%)	2 (6%)	0.79
Stable	57 (84%)	28 (80%)	29 (88%)	
Worsening	6 (9%)	4 (11%)	2 (6%)	
Change in subregions with BML V1 to V2 LFTJ (Category)				
Improvement	5 (7%)	3 (9%)	2 (6%)	0.83
Stable	62 (91%)	32 (91%)	30 (91%)	
Worsening	1 (1%)	0 (0%)	1 (3%)	
Change in subreiongs with BML V2 to V1—PFJ (Category)				
Improvement	13 (19%)	8 (23%)	5 (15%)	0.54
Stable	54 (79%)	27 (77%)	27 (82%)	
Worsening	1 (1%)	0 (0%)	1 (3%)	

V1: baseline visit; V2: 7 month visit; BML: bone marrow lesions; WB-EMS: whole body electromyostimulation

Fisher’s Exact test or Wilcoxon rank sum test was applied to calculate *p*-values

As shown in Table 3 very little osteophyte and meniscal change was seen over the observational period with only two participants showing any worsening in osteophytes and only one person showing worsening in meniscus pathology and extrusion each.

**Table 3:** Change in osteophytes and meniscus from V1 to V2

	**Overall** (*n* = 68)	**Control** (*n* = 35)	**WB-EMS** (*n* = 33)	***p*-value**
Osteophyte: Max change across all locations V1 to V2 Knee				
0	66 (97%)	34 (97%)	32 (97%)	1.00
1	2 (3%)	1 (3%)	1 (3%)	
Osteophyte: Max change across all locations V1 to V2 MFTJ				
0	66 (97%)	34 (97%)	32 (97%)	1.00
1	2 (3%)	1 (3%)	1 (3%)	
Osteophyte: Max change across all locations V1 to V2 LFTJ				
0	68 (100%)	35 (100%)	33 (100%)	1.00
	0 (0.0%)	0 (0.0%)	0 (0.0%)	
Osteophyte: Max change across all locations V1 to V2 PFJ				
0	68 (100%)	35 (100%)	33 (100%)	1.00
	0 (0.0%)	0 (0.0%)	0 (0.0%)	
Number of regions with any worsening in meniscus morphology V1 to V2—medial				
0	67 (99%)	34 (97%)	33 (100%)	1.00
1	1 (1%)	1 (3%)	0 (0%)	
Number of regions with any worsening in meniscus morphology V1 to V2—lateral				
0	68 (100%)	35 (100%)	33 (100%)	1.00
1	0 (0.0%)	0 (0.0%)	0 (0.0%)	
Change in meniscal extrusion V1 to V2—medial				
No	65 (98%)	35 (100%)	30 (97%)	0.47
Yes	1 (2%)	0 (0%)	1 (3%)	
Change in meniscal extrusion V1 to V2—lateral				
No	66 (100%)	34 (100%)	32 (100%)	1.00
Yes	0 (0%)	0 (0%)	0 (0%)	

V1: baseline visit; V2: 7 month visit; MFTJ -medial femoro-tibial joint; LFTJ: lateral femoro-tibial joint; PFJ: patello-femoral joint; WB-EMS: whole body electromyostimulation

Fisher’s Exact test or Wilcoxon rank sum test was applied to calculate *p*-values

In the WB-EMS group improvement in Hoffa synovitis was seen in 6% and in 9% in the CG, while for effusion-synovitis these numbers were 15% and 17%. Regarding worsening these numbers were 3% and 0% for Hoffa-synovitis and 12% and 9% for effusion synovitis (p for change of Hoffa-synovitis 0.83, for effusion-synovitis 0.78). The results for inflammatory MRI markers are shown in Table 4.

**Table 4:** Change in inflammatory markers from V1 to V2

	**Overall** (*n* = 68)	**Control** (*n* = 35)	**WB-EMS** (*n* = 33)	***p*-value**
MOAKS Hoffa-Synovitis—Change V1 to V2				
−1	5 (7%)	3 (9%)	2 (6%)	0.83
0	62 (91%)	32 (91%)	30 (91%)	
1	1 (1%)	0 (0%)	1 (3%)	
MOAKS Effusion-Synovitis—V1 to V2				
−2	1 (1%)	0 (0%)	1 (3%)	0.78
−1	10 (15%)	6 (17%)	4 (12%)	
0	50 (74%)	26 (74%)	24 (73%)	
1	7 (10%)	3 (9%)	4 (12%)	

V1: baseline visit; V2: 7 month visit; WB-EMS: whole body electromyostimulation

Fisher’s Exact test or Wilcoxon rank sum test was applied to calculate *p*-values

Concerning changes from V2 to V3, the only difference observed was that in the CG more knees showed worsening cartilage damage in the PFJ when compared to the EMS group. For all the other features no differences were observed. The details regarding changes from V2 to V3 are presented in supplementary Tables 7 to 11 in Appendix 3.

## Discussion

In this study focusing on structural change on the knee joint and compartmental level, embedded in a randomized control trial comparing WB-EMS training vs. standard of care over a 7-month period, we could show that in the WB-EMS group fewer subregions showed worsening cartilage damage on a whole knee level compared to the CG. However, no significant changes were observed for BMLs and changes in inflammatory markers. Further, very little change was seen for osteophytes, meniscal structure and meniscal extrusion without differences between groups. For almost all features assessed, no differences were seen between groups for changes from month 7 to month 12. The only difference observed between V2 and V3 was that more knees in the CG showed worsening of cartilage damage in the PFJ, a finding of unclear clinical relevance.

Our previous findings from the same cohort demonstrated that WB-EMS is highly effective in alleviating knee pain and improving function of the knee compared to a usual care group [16]. To date, only one other study has evaluated the effect of WB-EMS in individuals with knee OA. Park et al. examined the effectiveness of isometric strength exercise superimposed by WB-EMS compared to isometric exercises alone or a non-training control. In that study individuals with early knee OA (KL 1–2) were included while knee pain was not an eligibility criterion [15]. In addition, that study did not include MRI assessment.

Our group has evaluated intramuscular adipose tissue changes using MRI in another WB-EMS study not focusing on knee OA before [27]. We also assessed in a pilot study the spatial distribution of WB-EMS effects with respect to volume involvement and stimulation depth, using MRI-defined muscle edema as a marker of structural effects and found a heterogeneous pattern of edema attributed predominantly to different stimulus thresholds of the muscles and differences in the stress resistance of the muscles [28]. No studies looking at effects of WB-EMS have previously assessed joint structural changes over time with MRI.

There are limitations to our study. Structural OA was not uniformly defined radiologically as an inclusion criterion using the KL score. Since for reasons of time and economy, no application was made to the Federal Office for Radiation Protection for the acquisition of radiographs, we examined existing X-ray images and, if not available or too old, MRI was acquired. With this approach, the likelihood of including KL 0 and 1 knees or knees with end stage structural OA (KL 4) was minimized. We used the whole-organ MOAKS instrument to assess structural changes over time but not other MRI-based techniques to evaluate potential structural treatment effects. Future work should address whether more sensitive tools like segmentation-based cartilage assessment approaches may show more distinct differences between groups. The MRIs were read sequentially, and the reader was aware of the chronological order of images, a common approach in semi-quantitative MRI assessment of knee OA [29]. This might result in a slight tendency to read more change in comparison to a blinded reading. However, it has been shown that scoring without knowing the chronological sequence substantially decreases sensitivity in the detection of clinically relevant changes in comparison to scoring in chronological order and that it does not introduce false positive changes [30, 31].

Our observational period of 7 months was relatively short and OA being a slowly progressive disorder, not many structural changes on a joint level were expected or observed. For this reason, we included an extended follow-up visit at 12 months to understand whether there are post-interventional dynamics in regard to structural progression, which was not the case. In a previous study looking at effects of oral glucosamine over a 6 month period similarly little change in semi-quantitatively assessed structural parameters was observed [32].

Our sample size calculation did not focus on one of the outcomes presented here. Considering further the dichotomous nature of some data and the (however low) drop-out our sample size of factually 35 participants of the CG and 33 participants in the WB-EMS might be too low (i.e., underpowered) to address some of the outcomes of the present study.

According to various international guidelines, targeted physical training is a critical component of the treatment of knee OA. WB-EMS is a training approach that stimulates all the main muscle groups simultaneously, but each with dedicated intensity, and is known to have a wide range of positive health effects beyond knee OA. Due to its time efficiency, joint-friendliness and low subjective effort, WB-EMS training has the potential to reach a large target group of individuals with knee OA who are not receptive to physical training. For patients with pronounced pain and resulting kinesiophobia, WB-EMS could also be a promising way to reduce pain and build strength, thus creating an ideal basis for conventional land-based strength training.

In summary, we observed a difference with fewer subregions showing worsening cartilage damage on a whole knee level in the WB-EMS groups compared to the CG. However no significant changes were observed for changes in BMLs and changes in inflammatory markers. Very little change was seen for osteophytes and meniscal structure and extrusion. The finding of more knees in the CG showing cartilage worsening in the PFJ from month 7 to month 12 compared to the WB-EMS group remains elusive and should not be over-interpreted. The observed improvement in clinical outcome parameters in favor of the WB-EMS group is likely due to other effects than improvement or less worsening of joint structural changes.

## Supplementary Information

Below is the link to the electronic supplementary material.

ESM 1 (DOCX 20.7 KB)

ESM 2 (DOCX 15.7 KB)

ESM 3 (DOCX 25.1 KB)

## Data Availability

Data relative to this work will be available upon reasonable request to the corresponding author.
