# Supplementary material for: Structural effects of whole body electromyostimulation on knee osteoarthritis: the EMSOAT Study
Source: Skeletal Radiol. 2025 Jul 22;54(11):2579–88. doi: 10.1007/s00256-025-04984-5 (PMC12460362; doi:10.1007/s00256-025-04984-5)
Supplement: Supplementary file 1 — (DOCX 20.7 KB) [file 256_2025_4984_MOESM1_ESM.docx]

**Appendix 1 (a). Baseline cartilage damage –area extent**

|  | **Overall** | **Control** | **EMS** | **p-value** |
| --- | --- | --- | --- | --- |
| Cartilage area extent: Max score |  |  |  |  |
| 2 | 25 (37%) | 14 (40%) | 11 (33%) | 0.62 |
| 3 | 43 (63%) | 21 (60%) | 22 (67%) |  |
| Cartilage area extent: Number of SRs affected – mean (SD) | 7.6 (2.0) | 7.7 (2.1) | 7.5 (2.0) | 0.68 |
| Cartilage area extent: Max score MFTJ |  |  |  |  |
| 0 | 5 (7%) | 3 (9%) | 2 (6%) | 0.87 |
| 2 | 43 (63%) | 21 (60%) | 22 (67%) |  |
| 3 | 20 (29%) | 11 (31%) | 9 (27%) |  |
| Cartilage area extent: Number of SRs affected MFTJ |  |  |  |  |
| 0 | 5 (7%) | 3 (9%) | 2 (6%) | 0.19 |
| 1 | 4 (6%) | 4 (11%) | 0 (0%) |  |
| 2 | 12 (18%) | 4 (11%) | 8 (24%) |  |
| 3+ | 45 (69%) | 24 (69%) | 23 (70%) |  |
| Cartilage area extent: Max score LFTJ |  |  |  |  |
| 0 | 21 (31%) | 9 (26%) | 12 (36%) | 0.82 |
| 1 | 11 (16%) | 6 (17%) | 5 (15%) |  |
| 2 | 28 (41%) | 16 (46%) | 12 (36%) |  |
| 3 | 8 (12%) | 4 (11%) | 4 (12%) |  |
| Cartilage area extent: Number of SRs affected LFTJ |  |  |  |  |
| 0 | 21 (31%) | 9 (26%) | 12 (36%) | 0.45 |
| 1 | 21 (31%) | 10 (29%) | 11 (33%) |  |
| 2 | 10 (15%) | 5 (14%) | 5 (15%) |  |
| 3+ | 16 (24%) | 11 (35%) | 5 (15%) |  |
| Cartilage area extent: Max score PFJ |  |  |  |  |
| 0 | 1 (1%) | 1 (3%) | 0 (0%) | 1.00 |
| 1 | 2 (3%) | 1 (3%) | 1 (3%) |  |
| 2 | 41 (60%) | 21 (60%) | 20 (61%) |  |
| 3 | 24 (35%) | 12 (34%) | 12 (36%) |  |
| Cartilage area extent: Number of SRs affected PFJ |  |  |  |  |
| 0 | 1 (1%) | 1 (3%) | 0 (0%) | 0.81 |
| 1 | 5 (7%) | 3 (9%) | 2 (6%) |  |
| 2 | 12 (18%) | 7 (20%) | 5 (15%) |  |
| 3+ | 50 (74%) | 24 (69%) | 26 (79%) |  |

SRs: Subregions; Max: maximum; SD: standard deviation; MFTJ -medial femoro-tibial joint; LFTJ: lateral femoro-tibial joint; PFJ: patello-femoral joint; EMS: electromyostimulation

**Appendix 1 (b). Baseline cartilage damage – full thickness**

|  | **Overall** | **Control** | **EMS** | **p-value** |
| --- | --- | --- | --- | --- |
| Cartilage full-thickness: Max score |  |  |  |  |
| 0 | 2 (3%) | 2 (6%) | 0 (0%) | 0.51 |
| 1 | 6 (9%) | 2 (6%) | 4 (12%) |  |
| 2 | 48 (71%) | 24 (69%) | 24 (73%) |  |
| 3 | 12 (18%) | 7 (20%) | 5 (15%) |  |
| Cartilage full-thickness: Number SRs affected (mean- SD) | 3.5 (1.7) | 3.6 (1.9) | 3.4 (1.6) | 0.62 |
| Cartilage full-thickness: Max score MFTJ |  |  |  |  |
| 0 | 25 (37%) | 13 (37%) | 12 (36%) | 0.92 |
| 1 | 10 (15%) | 6 (17%) | 4 (12%) |  |
| 2 | 31 (46%) | 15 (43%) | 16 (48%) |  |
| 3 | 2 (3%) | 1 (3%) | 1 (3%) |  |
| Cartilage full-thickness: Number SRs affected MFTJ |  |  |  |  |
| 0 | 25 (37%) | 13 (37%) | 12 (36%) | 1.00 |
| 1 | 15 (22%) | 7 (20%) | 8 (24%) |  |
| 2 | 13 (19%) | 7 (20%) | 6 (18%) |  |
| 3+ | 15 (22%) | 8 (23%) | 7 (21%) |  |
| Cartilage full-thickness: Max score LFTJ |  |  |  |  |
| 0 | 45 (66%) | 21 (60%) | 24 (73%) | 0.60 |
| 1 | 11 (16%) | 7 (20%) | 4 (12%) |  |
| 2 | 8 (12%) | 4 (11%) | 4 (12%) |  |
| 3 | 4 (6%) | 3 (9%) | 1 (3%) |  |
| Cartilage full-thickness: Number SRs affected LFTJ |  |  |  |  |
| 0 | 45 (66%) | 21 (60%) | 24 (73%) | 0.09 |
| 1 | 12 (18%) | 6 (17%) | 6 (18%) |  |
| 2 | 5 (7%) | 5 (14%) | 0 (0%) |  |
| 3+ | 6 (9%) | 3 (9%) | 3 (9%) |  |
| Cartilage full-thickness: Max score PFJ |  |  |  |  |
| 0 | 11 (16%) | 7 (20%) | 4 (12%) | 0.83 |
| 1 | 26 (38%) | 12 (34%) | 14 (42%) |  |
| 2 | 25 (37%) | 13 (37%) | 12 (36%) |  |
| 3 | 6 (9%) | 3 (9%) | 3 (9%) |  |
| Cartilage full-thickness: Number SRs affected PFJ |  |  |  |  |
| 0 | 11 (16%) | 7 (20%) | 4 (12%) | 0.84 |
| 1 | 23 (34%) | 11 (31%) | 12 (36%) |  |
| 2 | 25 (37%) | 12 (34%) | 13 (39%) |  |
| 3 | 9 (13%) | 5 (14%) | 4 (12%) |  |

SRs: Subregions; Max: maximum; SD: standard deviation; MFTJ -medial femoro-tibial joint; LFTJ: lateral femoro-tibial joint; PFJ: patello-femoral joint; EMS: electromyostimulation
