# Supplementary material for: Structural effects of whole body electromyostimulation on knee osteoarthritis: the EMSOAT Study
Source: Skeletal Radiol. 2025 Jul 22;54(11):2579–88. doi: 10.1007/s00256-025-04984-5 (PMC12460362; doi:10.1007/s00256-025-04984-5)
Supplement: Supplementary file 2 — (DOCX 15.7 KB) [file 256_2025_4984_MOESM2_ESM.docx]

**Appendix 2. BML worsening (maximum increase / delta) from V1 to V2**

| **Change in BML** | **Overall** | **Control** | **EMS** | **p-value** |
| --- | --- | --- | --- | --- |
| Maximum increase in BML score V1 to V2 - Knee |  |  |  |  |
| 0 | 42 (62%) | 19 (54%) | 23 (70%) | 0.43 |
| 1 | 23 (34%) | 14 (40%) | 9 (27%) |  |
| 2 | 3 (4%) | 2 (6%) | 1 (3%) |  |
| Maximum increase in BML score > 0 - V1 to V2 -Knee |  |  |  |  |
| No | 42 (62%) | 19 (54%) | 23 (70%) | 0.22 |
| Yes | 26 (38%) | 16 (46%) | 10 (30%) |  |
| Maximum increase in BML score V1 to V2 - MFTJ |  |  |  |  |
| 0 | 50 (74%) | 25 (71%) | 25 (76%) | 1.00 |
| 1 | 15 (22%) | 8 (23%) | 7 (21%) |  |
| 2 | 3 (4%) | 2 (6%) | 1 (3%) |  |
| Maximum increase in BML score > 0 - V1 to V2 - MFTJ |  |  |  |  |
| No | 50 (74%) | 25 (71%) | 25 (76%) | 0.79 |
| Yes | 18 (26%) | 10 (29%) | 8 (24%) |  |
| Maximum increase in BML score V1 to V2 - LFTJ |  |  |  |  |
| 0 | 62 (91%) | 31 (89%) | 31 (94%) | 0.36 |
| 1 | 5 (7%) | 4 (11%) | 1 (3%) |  |
| 2 | 1 (1%) | 0 (0%) | 1 (3%) |  |
| Maximum increase in BML score > 0 - V1 to V2 - LFTJ |  |  |  |  |
| No | 62 (91%) | 31 (89%) | 31 (94%) | 0.67 |
| Yes | 6 (9%) | 4 (11%) | 2 (6%) |  |
| Maximum increase in BML score V1 to V2 - PFJ |  |  |  |  |
| 0 | 63 (93%) | 31 (89%) | 32 (97%) | 0.36 |
| 1 | 5 (7%) | 4 (11%) | 1 (3%) |  |

V1: baseline visit; V2: 7 month visit; MFTJ -medial femoro-tibial joint; LFTJ: lateral femoro-tibial joint; PFJ: patello-femoral joint; EMS: electromyostimulation
