# Supplementary material for: Structural effects of whole body electromyostimulation on knee osteoarthritis: the EMSOAT Study
Source: Skeletal Radiol. 2025 Jul 22;54(11):2579–88. doi: 10.1007/s00256-025-04984-5 (PMC12460362; doi:10.1007/s00256-025-04984-5)
Supplement: Supplementary file 3 — (DOCX 25.1 KB) [file 256_2025_4984_MOESM3_ESM.docx]

**APPENDIX 3.**

**Table 1 Suppl. Cartilage subregion worsening V2 to V3
(area extent and/or full thickness dimensions including within-grade changes)**

|  | **Overall** | **Control** | **EMS** | **p-value** |
| --- | --- | --- | --- | --- |
| Number of subregions with worsening (including within-grade changes) V2 to V3 – Knee | | | | |
| 0 | 44 (71%) | 18 (60%) | 26 (81%) | 0.10 |
| 1 | 14 (23%) | 8 (27%) | 6 (19%) |  |
| 2 | 3 (5%) | 3 (10%) | 0 (0%) |  |
| 3 | 1 (2%) | 1 (3%) | 0 (0%) |  |
| Any subregions with worsening (including within-grade changes) V2 to V3 – Knee | | | | |
| No | 44 (71%) | 18 (60%) | 26 (81%) | 0.09 |
| Yes | 18 (29%) | 12 (40%) | 6 (19%) |  |
| Number of subregions with worsening (including within-grade changes) V2 to V3 – MFTJ | | | | |
| 0 | 52 (84%) | 25 (83%) | 27 (84%) | 0.86 |
| 1 | 9 (15%) | 4 (13%) | 5 (16%) |  |
| 2+ | 1 (2%) | 1 (3%) | 0 (0%) |  |
| Any subregions with worsening (including within-grade changes) V2 to V3 – MFTJ | | | | |
| No | 52 (84%) | 25 (83%) | 27 (84%) | 1.00 |
| Yes | 10 (16%) | 5 (17%) | 5 (16%) |  |
| Number of subregions with worsening (including within-grade changes) V2 to V3 – LFTJ | | | | |
| 0 | 57 (92%) | 26 (87%) | 31 (97%) | 0.19 |
| 1+ | 5 (8%) | 4 (13%) | 1 (3%) |  |
| Number of subregions with worsening (including within-grade changes) V2 to V3 – PFJ | | | | |
| 0 | 56 (90%) | 24 (80%) | 32 (100%) | 0.01 |
| 1 | 5 (8%) | 5 (17%) | 0 (0%) |  |
| 2+ | 1 (2%) | 1 (3%) | 0 (0%) |  |
| Any subregions with worsening (including within-grade changes) V2 to V3 – PFJ | | | | |
| No | 56 (90%) | 24 (80%) | 32 (100%) | 0.01 |
| Yes | 6 (10%) | 6 (20%) | 0 (0%) |  |

**Table 2 suppl. Change in bone marrow lesions (BMLs) – subregion approach V2 to V3**

|  | **Overall** | **Control** | **EMS** | **p-value** |
| --- | --- | --- | --- | --- |
| Change in subregions with BML V2 to V3 - Knee | | | | |
| Improvement | 32 (52%) | 14 (47%) | 18 (56%) | 0.68 |
| Stable | 24 (39%) | 13 (43%) | 11 (34%) |  |
| Worsening (1) | 5 (8%) | 2 (7%) | 3 (9%) |  |
| Worsening (2+) | 1 (2%) | 1 (3%) | 0 (0%) |  |
| Change in subregions with BML V2 to V3 - MFTJ (Category) | | | | |
| Improvement | 23 (37%) | 15 (50%) | 8 (25%) | 0.07 |
| Stable | 35 (56%) | 13 (43%) | 22 (69%) |  |
| Worsening (1) | 3 (5%) | 1 (3%) | 2 (6%) |  |
| Worsening (2+) | 1 (2%) | 1 (3%) | 0 (0%) |  |
| Change in subregions with BML V2 to V3 LFTJ (Category) | | | | |
| Improvement | 8 (13%) | 4 (13%) | 4 (13%) | 0.55 |
| Stable | 52 (84%) | 24 (80%) | 28 (88%) |  |
| Worsening (1) | 2 (3%) | 2 (7%) | 0 (0%) |  |
| Change in subreiongs with BML V2 to V3 - PFJ (Category) | | | | |
| Improvement | 12 (19%) | 5 (17%) | 7 (22%) | 0.84 |
| Stable | 45 (73%) | 22 (73%) | 23 (72%) |  |
| Worsening (1) | 5 (8%) | 3 (10%) | 2 (6%) |  |

**Table 3 Suppl. BML worsening (maximum increase / delta from V2 to V3)**

| **Change in BML** | **Overall** | **Control** | **EMS** | **p-value** |
| --- | --- | --- | --- | --- |
| Maximum increase in BML score V2 to V3 - Knee |  |  |  |  |
| 0 | 33 (53%) | 14 (47%) | 19 (59%) | 0.80 |
| 1 | 21 (34%) | 12 (40%) | 9 (28%) |  |
| 2 | 6 (10%) | 3 (10%) | 3 (9%) |  |
| 3 | 2 (3%) | 1 (3%) | 1 (3%) |  |
| Maximum increase in BML score > 0 – V2 to V3 -Knee |  |  |  |  |
| No | 33 (53%) | 14 (47%) | 19 (59%) | 0.45 |
| Yes | 29 (47%) | 16 (53%) | 13 (41%) |  |
| Maximum increase in BML score V2 to V3 - MFTJ |  |  |  |  |
| 0 | 49 (79%) | 23 (77%) | 26 (81%) | 0.62 |
| 1 | 10 (16%) | 6 (20%) | 4 (13%) |  |
| 2 | 3 (5%) | 1 (3%) | 2 (6%) |  |
| Maximum increase in BML score > 0 – V2 to V3 - MFTJ |  |  |  |  |
| No | 49 (79%) | 23 (77%) | 26 (81%) | 0.76 |
| Yes | 13 (21%) | 7 (23%) | 6 (19%) |  |
| Maximum increase in BML score V2 to V3 - LFTJ |  |  |  |  |
| 0 | 52 (84%) | 23 (77%) | 29 (91%) | 0.21 |
| 1 | 7 (11%) | 4 (13%) | 3 (9%) |  |
| 2 | 3 (5%) | 3 (10%) | 0 (0%) |  |
| Maximum increase in BML score > 0 – V2 to V3 - LFTJ |  |  |  |  |
| No | 52 (84%) | 23 (77%) | 29 (91%) | 0.18 |
| Yes | 10 (16%) | 7 (23%) | 3 (9%) |  |
| Maximum increase in BML score V2 to V3 - PFJ |  |  |  |  |
| 0 | 48 (77%) | 23 (77%) | 25 (78%) | 1.00 |
| 1 | 14 (23%) | 7 (23%) | 7 (22%) |  |

**Table 4 suppl. Change in osteophytes and meniscus – V2 to V3**

|  | **Overall** | **Control** | **EMS** | **p-value** |
| --- | --- | --- | --- | --- |
| Osteophyte: Max change across all locations V2 to V3 Knee | | | | |
| 0 | 53 (85%) | 25 (83%) | 28 (88%) | 0.73 |
| 1 | 9 (15%) | 5 (17%) | 4 (13%) |  |
| Osteophyte: Max change across all locations V2 to V3 MFTJ | | | | |
| 0 | 53 (85%) | 25 (83%) | 28 (88%) | 0.48 |
| 1 | 9 (15%) | 5 (17%) | 4 (13%) |  |
| Osteophyte: Max change across all locations V2 to V3 LFTJ | | | | |
| 0 | 58 (94%) | 28 (93%) | 30 (94%) | 1.00 |
|  | 4 (6%) | 2 (7%) | 2 (6%) |  |
| Osteophyte: Max change across all locations V2 to V3 PFJ | | | | |
| 0 | 57 (92%) | 28 (93%) | 29 (91%) | 1.00 |
|  | 5 (8%) | 2 (7%) | 3 (9%) |  |
| Number of regions with any worsening in meniscus morphology V2 to V3 - medial | | | | |
| 0 | 61 (98%) | 30 (100%) | 31 (97%) | 1.00 |
| 1 | 1 (2%) | 0 (0%) | 1 (3%) |  |
| Number of regions with any worsening in meniscus morphology V2 to V3 - lateral | | | | |
| 0 | 61 (98%) | 30 (100%) | 31 (97%) | 1.00 |
| 1 | 1 (2%) | 0 (0%) | 1 (3%) |  |

**Table 5 suppl. Change in inflammatory markers V2 to V3**

|  | **Overall** | **Control** | **EMS** | **p-value** |
| --- | --- | --- | --- | --- |
| MOAKS Hoffa-Synovitis - Change V2 to V3 | | | | |
| -1 | 5 (8%) | 2 (7%) | 3 (9%) | 0.87 |
| 0 | 54 (87%) | 26 (87%) | 28 (88%) |  |
| 1 | 3 (5%) | 2 (7%) | 1 (3%) |  |
| MOAKS Effusion-Synovitis – V2 to V3 | | | | |
| -1 | 8 (13%) | 6 (20%) | 2 (6%) | 0.24 |
| 0 | 40 (65%) | 19 (63%) | 21 (66%) |  |
| 1 | 14 (23%) | 5 (17%) | 9 (28%) |  |
